# Posttransplant maintenance therapy in multiple myeloma: the changing landscape

**DOI:** 10.1038/bcj.2017.23

**Published:** 2017-03-24

**Authors:** S Sengsayadeth, F Malard, B N Savani, L Garderet, M Mohty

**Affiliations:** 1 Section of Hematology and Stem Cell Transplant, Vanderbilt Ingram Cancer Center, Nashville, TN, USA; 2 Department of Haematology, Saint Antoine Hospital, University Pierre and Marie Curie, and INSERM UMRs 938, Paris, France

## Abstract

Transplant-eligible patients with multiple myeloma (MM) now have extended survival after diagnosis owing to effective modern treatment strategies that include new agents in induction therapy, autologous stem cell transplant (ASCT), consolidation therapy and posttransplant maintenance therapy. Standard of care for newly diagnosed, fit patients includes ASCT and, often nowadays, posttransplant maintenance. Several large studies have shown the efficacy of maintenance with thalidomide, lenalidomide and bortezomib in the treatment scheme of MM with regards to prolonging progression-free survival and, to a lesser degree, overall survival. Herein we discuss the data currently available to support the use of maintenance therapy in patients after ASCT as well as the newer available agents that may be a part of its changing landscape in the years to come.

## Introduction

In the past two decades, multiple myeloma (MM) has emerged from being an almost uniformly fatal hematological malignancy to one for which there is now a major arsenal of transformative new therapies. Before the turn of the century, the median survival after diagnosis for patients requiring therapy was approximately 3 years.^1^ In the current era of modern treatment, the median survival after induction chemotherapy and autologous stem cell transplant (ASCT) alone reached 7 years.^1, 2, 3^ High-dose chemotherapy with melphalan followed by ASCT remains one of the mainstays of treatment and is considered the standard of care for fit, eligible patients.^4, 5, 6, 7, 8^ Despite good response rates with ASCT with duration of response up to years after the procedure, relapses are almost inevitable for most patients, primarily due to incomplete eradication of residual myeloma cells. It is postulated that the depth of response to primary therapy is associated with improved outcomes, particularly in the setting of ASCT.^9^ Furthermore, achievement of complete response and in particular a minimal residual disease-negative state after autologous hematopoietic stem cell transplantation may translate into improved progression-free survival (PFS) and even overall survival (OS).^10, 11, 12, 13, 14^ Two different approaches have been developed to pursue treatment after induction therapy: consolidation and maintenance therapy. Consolidation is a short treatment, generally consisting of a single agent or combination therapy or a second autologous hematopoietic stem cell transplantation aimed to increase the depth of the response. Maintenance therapy is applied for a longer period, usually for 2–3 years or even until disease progression, in order to maintain the depth of the response. Per definition, maintenance must be a long-term treatment, therefore for the purpose of this review we defined posttransplant maintenance as treatment administered for at least 1 year. Maintenances administered <1 year should probably be considered as consolidation treatment and are not addressed in this review.

Although first maintenance attempts with conventional chemotherapy, steroids or interferon-alpha were disappointing,^15, 16, 17, 18, 19, 20^ in recent years posttransplant maintenance using new agents—thalidomide, lenalidomide, bortezomib—to augment the posttransplant response and its duration has arguably been part of the standard of care for many patients after ASCT.^9^ Ideal agents for maintenance are those that are easily administered (for many, this entails being an oral drug) and induce minimal toxicity while maintaining the initial response to upfront therapy. Herein we look at the current data regarding maintenance therapy and discuss the newer emerging agents that may be incorporated in future posttransplant treatment strategies.

## Thalidomide maintenance

Thalidomide was the first of the novel drugs belonging to the class of immunomodulatory agents (IMIDs) to garner interest as potential post-ASCT maintenance. In the late 1990s, early phase studies showed that thalidomide antiangiogenesis properties had significant antitumor effects in MM,^21^ and since then, thalidomide has been incorporated in induction regimens.^22, 23^ Thereafter, several studies, as outlined in Table 1, have shown improvement in PFS and OS, albeit the latter to a lesser degree, when thalidomide is incorporated into maintenance therapy post-ASCT.^2, 24, 25, 26, 27, 28, 29^ Thalidomide, however, is associated with significant and often dose-limiting toxicity, with the predominant adverse effects being peripheral neuropathy and an increased risk for venous thromboembolic events. There are also data suggesting that thalidomide maintenance should not be offered to patients with poor-risk cytogenetics as determined by fluorescence *in situ* hybridization, as these patients have been shown to have inferior outcome compared with controls.^26^ Meta-analyses of thalidomide maintenance by Morgan *et al.*^26^ and Kayoga *et al.*^30^ have shown a significant OS benefit (*P*<0.001, hazard ratio (HR)=12.3; 95% confidence interval (CI), 5.5–19.0) and improved PFS (HR=0.65, *P*<0.01) and OS (HR=0.83, *P*=0.07), respectively. In clinical practice, however, long-term use of thalidomide is often limited by its toxicity. Additionally, there are some data to suggest that there may an increased risk of secondary primary malignancies (SPMs) as well.^28^

## Lenalidomide maintenance

Lenalidomide, a less toxic and more potent derivative of thalidomide with similar immunomodulatory effects, has been shown to be active in relapsed or refractory MM.^31^ Similarly to its parent drug, it has since been studied and shown to be effective in the front line^32, 33, 34^ and maintenance MM treatment setting.^35, 36, 37^ Owing to its better tolerability than thalidomide, it is now the preferred IMID for maintenance treatment. This is based on several large trials that have shown its efficacy post-ASCT (Table 2).

Attal *et al.*^35^ and McCarthy *et al.*^36^ both described the use of lenalidomide maintenance post-ASCT as well tolerated and associated with statistically significant improved PFS. Attal *et al.*^35^ reported in the Intergroupe Francophone du Myeloma (IFM) study 614 patients aged <65 years of age who underwent ASCT, lenalidomide consolidation therapy (25 mg per day on days 1–21 of 28-day cycle for 2 cycles) and were treated with either lenalidomide maintenance (10 mg for 3 months, followed by increase in dosing to 15 mg if tolerated until relapse) or placebo maintenance. Median PFS was significantly improved in the lenalidomide maintenance group, being 41 months, as compared with 23 months in the placebo group (HR: 0.50, *P*<0.0001). At 70%, OS was similar at 4 years in both groups, though the authors note that their study was not powered to detect a difference in survival.^35^ The Cancer and Leukemia Group B (CALGB) Trial reported by McCarthy *et al.*^36^ similarly showed an improvement in PFS in their study of 460 patients aged <71 years. Maintenance therapy with lenalidomide was started at 100 days after ASCT at 10 mg daily dosing until disease progression. After a median follow-up of 34 months, they observed a median PFS of 46 months in the lenalidomide group compared with 27 months in the placebo group (*P*<0.001), and a 3-year PFS also favoring the lenalidomide group (58 vs 37%, HR=0.48, 95% CI, 0.36–0.63). In contrast to the IFM study, the latter did detect a better OS in the lenalidomide group (88%, 95% CI, 84–93 vs 80% (HR 0.62, 95% CI, 0.40–0.95, *P*=0.03)).^36^ There were some differences between the two trial protocols that could have impacted the reported outcomes. The CALGB trial induction treatments included novel agents, including thalidomide and lenalidomide, while the IFM trial induction included vincristine-based therapy plus or minus adriamycin. Neither postinduction therapy prior to ASCT nor post-ASCT consolidation therapy was given in the CALGB trial, while all patient received two cycles of lenalidomide consolidation in the IFM trial. Also notably, whereas no crossover was allowed in the IFM trial, crossover was allowed in the CALBG study. However, it should be noted that crossover was not allowed in placebo patients who had not progressed.^35, 36^

Similarly to the IFM study, Palumbo *et al.*^37^ reported in the Gruppo Italiano Malattie Emotologiches dell'Adulto (GIMEMA) trial that 273 transplant-eligible patients aged <65 years and who received lenalidomide maintenance, 10 mg on days 1–21 of each 28-day cycle until disease progression, after either ASCT or melphalan, prednisone and lenalidomide consolidation (MPR) had an improvement in PFS (median PFS 41.9 vs 21 months in the treatment group, HR=0.47, 95% CI, 0.33–0.65, *P*<0.001) but not in OS (88.0 vs 79.2%, HR=0.64, 95% CI, 0.36–1.15, *P*=0.14). In their study, the clinical benefit of lenalidomide maintenance therapy was independent of the type of consolidation (ASCT or MPR), and the response rates improved with maintenance, with relapse being delayed by approximately 2 years.^37^

Lenalidomide maintenance appears to be better tolerated than thalidomide with regards to severe, dose-limiting side effects. The most common adverse effects were hematological (specifically neutropenia) as well as dermatological. Though less common, the most striking adverse event noted in these clinical trials was the development of SPMs in patients who received lenalidomide maintenance. Both the IFM and CALGB studies showed increased risk of hematological and non-hematological second malignancies (2–3 fold increased risk), whereas Palumbo *et al.*^37^ noted no difference in SPMs. This increased risk warrants discussion with all patients who undertake maintenance therapy as part of their treatment plan post-ASCT.^35, 36, 37^

Although the benefits of lenalidomide maintenance after ASCT clearly outweigh the small risk of SPMs, some argue that the lack of proof of OS benefit within these clinical trials makes it arguable as to whether lenalidomide maintenance should be considered the standard of care after transplant. However, a recent meta-analysis involving 1209 patients within these 3 major randomized clinical trials of lenalidomide maintenance after ASCT (CALGB, IFM, GIMEMA) demonstrates a significantly prolonged OS compared with controls in patients with MM in all response categories.^3^ With a median follow-up of 6.6 years, median OS for patients who received lenalidomide maintenance was not reached compared with 82 months in the control group (HR=0.74; 95% CI, 0.62–0.89; log-rank *P*=0.001) and 5-, 6- and 7-year OS were longer in the maintenance therapy group (71 vs 66%, 65 vs 58% and 62 vs 50%, respectively). These data strongly suggest that lenalidomide maintenance should be discussed post-ASCT in all patients in whom it is tolerated as part of the standard of care.^3^ However, we must keep in mind that, at the moment, lenalidomide is not approved by the Food and Drug Administration (FDA) and European Medicines Agency (EMA).

## Bortezomib maintenance

Bortezomib, the first novel agent in the class of proteasome inhibitors, has been shown in early trials to have significant activity in MM.^39^ Like the IMIDs, it has since been integrated in standard induction therapy for many newly diagnosed patients, including those with renal impairment.^23, 40, 41, 42, 43^ Bortezomib garnered FDA and EMA approval for relapsed/refractory disease based on the APEX trial^44^ and, eventually, in 2008 for newly diagnosed disease.

In the post-ASCT setting, there are two major trials that have evaluated the efficacy of bortezomib (Table 3). The phase III HOVON-65/GMMG-HD4 Trial, reported by Sonneveld *et al.*^41^ compared vincristine, adriamycin and dexamethasone (VAD) induction vs bortezomib, adriamycin and dexamethasone (PAD) followed by ASCT and maintenance therapy consisting of either thalidomide 50 mg daily in the VAD group or bortezomib 1.3 mg/m^2^ every 2 weeks for 2 years in the PAD group. This study has been recently updated,^45^ with a median follow-up of 91.4 months, the PFS in the PAD group was superior to that in the VAD group (34 vs 28 months, HR=0.77, 95% CI, 0.65–0.90, *P*=0.001). The median OS was 90 months in the PAD group compared with 83 months in the VAD group, and the restricted mean survival time was 4.8 months (95% CI, 0.2–9.5, *P*=0.04) in favor of the PAD group. In the subgroup analyses of patients with renal impairment (baseline creatinine of >2 mg/dl), the 5-year PFS was higher in the PAD group (32 vs 5%, *P*=0.001) as was the 5-year OS (66 vs 21%, *P*<0.001).^45, 46^ The PFS was similar for patients who had baseline creatinine ⩽2 or>2 mg/dl, leading the investigators to conclude that use of bortezomib before ASCT and as posttransplant maintenance may overcome the negative prognostic impact of renal impairment in newly diagnosed transplant-eligible patients.^46^ Another analysis of the same study showed rather strikingly that patients with deletion 17p (del17p13) derived the most benefit from bortezomib-containing regimens.^47^ In an update, both 5-year PFS and OS were superior using the bortezomib-containing regimens, 22% and 65%, respectively, compared with non-bortezomib-containing regimens, 5% and 18%, respectively.^45^ These data show that bortezomib treatment significantly improved PFS and OS in patients with del 17p, suggesting that the known adverse impact of del 17p on PFS and OS can be markedly reduced by incorporating bortezomib into the treatment and may be important for the long-term management of patients with this high-risk feature.

Rosinol *et al.*^43^ from the Spanish Myeloma group published on 386 patients who received maintenance therapy 3 months after induction and ASCT with (1) interferon-alpha 2b 3 million units subcutaneously thrice weekly, (2) thalidomide 100 mg daily or (3) thalidomide 100 mg daily plus bortezomib 1.3 mg/m^3^ on days 1, 4, 8 and 11 every 3 months for up to 3 years. With a median follow-up of 24 months, an improved 2-year PFS was seen in the thalidomide+bortezomib group vs the thalidomide alone and the interferon alpha-2b groups (78 vs 63 vs 49%, *P*=0.01). No difference in OS was detected.^43^

## Summary of available trials

Most of the data to support maintenance therapy in the after autologous stem cell setting are related to the use of the oral IMIDS, thalidomide and lenalidomide. Long-term thalidomide therapy, however, appears to be limited by its significant side effect profile, which includes dose-limiting peripheral neuropathy requiring cessation of therapy in a significant number of patients. Lenalidomide, its more potent analog, has several large phase III studies and a recent meta-analysis strongly supporting its use in post-ASCT maintenance. It has been shown to delay progression by approximately 2 years as well as have a significant OS benefit.^3, 35, 36, 37^ However, the concern with lenalidomide in the maintenance setting has been the increased risk of SPMs in patients who received lenalidomide compared with placebo, which is an important consideration particularly in the setting of most patients having prior exposure to high-dose melphalan therapy. The proteasome inhibitor bortezomib is also well tolerated after transplant and may be preferred in the event of renal impairment and high-risk features, such as the 17p deletion. No increased risk of SPMs was seen in bortezomib trials, but its injectable form could be a limit for long-term maintenance therapy. However, subcutaneous administration is well tolerated^48^ and home administration is feasible^49^ and could further improve patients' acceptance, particularly in the setting of a long-term maintenance therapy. Recently, an oral proteasome inhibitors, namely ixazomib, have emerged as efficacious for MM in the relapsed, refractory setting^50^ that is also being investigated for maintenance use.

There are limited data on the impact of long-term maintenance therapy on the quality of life in patients with MM, and this needs to be further investigated in future trials. Stewart *et al.*^28^ did note in their study that long-term maintenance with thalidomide appeared to adversely impact patient's quality of life and much of it was attributed to the side effect profile of the drug itself.

## Newer agents

### Proteasome inhibitors

Ixazomib is a new oral, second-generation peptide boronic acid proteasome inhibitor that acts similarly to bortezomib in that it targets nuclear factor-κB and has antiangiogenic properties^51^ and has been shown to have strong preclinical efficacy.^52, 53^ Similarly to its IMID counterparts, one of the attractive features of ixazomib is its oral administration. It has been shown in early phase I/II trials, as part of an all oral induction regimen of ixazomib, lenalidomide and dexamethasone, to be well tolerated and active as upfront treatment for newly diagnosed patients.^54^ Recently, a large double-blind, placebo-controlled phase III clinical trial with 722 patients was published that demonstrated improved PFS in patients with relapsed/refractory disease, which led to the FDA approval for this indication. Patients who received ixazomib in addition to lenalidomide and dexamethasone had a PFS of 20.6 months compared with just 14 months (HR 0.74, *P*=0.01) for those who received lenalidomide and dexamethasone alone. This difference was seen in all subgroups of patients, including those with high-risk cytogenetics. At 23 months, the median OS was not reached in either group. In patients who received ixazomib, there was limited additional toxicity with patient-reported quality of life similar in both groups.^50^ Several clinical trials are ongoing investigating its use alone or in combination with lenalidomide in the posttransplant maintenance setting (Table 4).

Carfilzomib is a next-generation irreversible proteasome inhibitor that binds selectively and irreversibly to the constitutive proteasome and immunoproteasome. Carfilzomib is equally potent but more selective for the chymotrypsine-like activity of the proteasome than bortezomib and preclinical data have shown efficacy of carfilzomib in hematological malignancies, with a higher cytotoxicity compared with bortezomib.^55^ Carfilzomib monotherapy has therefore been shown to be effective in relapse MM in early phase I/II trial.^56, 57^ Recently, a large randomized phase III clinical trial with 792 patients reported that the addition of carfilzomib to the combination of lenalidomide and dexamethasone improved PFS in patients with relapsed/refractory disease, which led to the FDA and EMA approval for this indication.^58^ Carfilzomib is now being evaluated as part of the combination therapy for first-line MM treatment in patients eligible to ASCT. Bringhen *et al.*^59^ recently reported the results of a phase I/II study evaluating weekly carfilzomib, cyclophosphamide and dexamethasone followed by maintenance with weekly carfilzomib in elderly patients with MM. Carfilzomib maintenance appears to be safe and effective with an improvement of complete remission rate from 12% to 40%. Overall, results from carfilzomib use in relapse/refractory MM and in the non-transplant maintenance setting suggest that posttransplant maintenance therapy using carfilzomib may be relevant, despite its intravenous administration. Carfilzomib is currently evaluated in this setting in several clinical trials (Table 4).

## Monoclonal antibodies

Monoclonal antibodies directed against targets expressed on myeloma cells have emerged as an effective treatment for MM. Among others, elotuzumab, which targets SLAMF-7, and daratumumab, which targets CD38, are the monoclonal antibodies with the most advanced clinical development. Elotuzumab and daratumumab are approved by the FDA and EMA for relapsed/refractory MM. A large randomized phase III clinical trial with 646 patients recently reported that the addition of elotuzumab to lenalidomide and dexamethasone in relapse /refractory MM was associated with an improved PFS (19.4 vs 14.9 months in the control group; *P*<0.001).^60^ Regarding daratumumab, two phase I/II studies reported that the use of daratumumab monotherapy in relapsed/refractory MM was associated with overall response rate of 36% and 29.2% respectively.^61, 62^ More recently, two large randomized phase III studies evaluated the addition of daratumumab to the combination of bortezomib plus dexamethasone^63^ or lenalidomide and dexamethasone^64^ in relapse/refractory MM. Both studies have shown that the addition of daratumumab improved PFS, time to progression and overall response rate.^63, 64^ Elotuzumab and daratumumab are now being evaluated for MM first-line treatment. A prospective randomized phase III study is ongoing evaluating the combination of bortezomib, thalidomide and dexamethasone with or without daratumumab, before ASCT, followed by a maintenance therapy with daratumumab (NCT025411383). Similarly, elotuzumab-based maintenance after ASCT is currently investigated in several clinical trials (Table 4).

### Histone deacetylase inhibitors (HDAC-I)

HDAC-I have recently been shown to be effective in treatment for MM. Their mechanism of action is via targeting of epigenetic-silencing mechanisms, increasing the susceptibility of tumor cells to immune-mediated killing and inhibition of cytokine release to disrupt the tumor microenvironment.^65, 66, 67, 68^ Several trials have since shown the efficacy of the non-specific HDAC-I, vorinostat, in combination with conventional drug therapy in relapsed/refractory MM.^68, 69, 70, 71, 72^ Recently, a phase I study showed the tolerability of the combination of lenalidomide and vorinostat as post-ASCT maintenance therapy for MM, which showed an improvement in posttransplant response in 7 out of the 16 patients.^73^

Another HDAC-I, panobinostat, has also shown to have efficacy in preclinical studies.^74, 75^ A large phase III clinical trial demonstrated that, when panobinostat was added to bortezomib and dexamethasone, it led to improvements in PFS^76^ as well as induced responses in heavily pretreated bortezomib-refractory patients.^77^ There are minimal data in the posttransplant maintenance setting, although a series of cases has been reported using panobinostat as a maintenance drug in patients with relapsed disease.^78^

Both HDAC-I drugs are attractive options due to their oral bioavailability and appear to be well tolerated in early clinical trials. Further investigations are ongoing to ascertain their role in the posttransplant maintenance setting (Table 4).

## Summary

Maintenance therapy in MM is an integral part of improving patient outcome after autologous hematopoietic stem cell transplantation due to its role in suppression of residual disease. ASCT remains the standard of care for fit, eligible, newly diagnosed patients after induction therapy. Although, its wider use has been tempered by the recognition of a small increased risk of SPMs, the results of the most recent meta-analysis showing a significant OS benefit in patients treated with lenalidomide maintenance argue in favor of establishing lenalidomide maintenance as the standard of care in the post-ASCT setting, provided approval is granted by the FDA and EMA.^3^ Similarly, large trial data support the use of bortezomib use in posttransplant maintenance, particularly in high-risk patients.

Second-generation proteasome inhibitors, monoclonal antibodies and HDAC-I provides the scope for them to be studied in the posttransplant maintenance setting, particularly in relation to tolerability, and whether serious side effects such as SPMs are part of their profile. Oral agents, including ixazomib, vorinostat and panobinostat, are particularly interesting in this setting. As patients are living longer with MM, strategies to prolong their survival along with ensuring good quality of life are important in optimizing patient care.

## Figures and Tables

**Table 1:** Major studies of thalidomide maintenance

*Study*	N	*Regimen*	*Duration*	*PFS*	*OS*
Attal *et al.*^23^	597	No maintenance vs pamidronate vs pamidronate+Thal 400 mg	15 months median for Thal	36 vs 37 vs 52% (*P*<0.009)	77 vs 74 vs 87% (*P*<0.04)
Barlogie *et al.*^2, 24^	668	No Thal vs Thal maintenance 100 mg daily for first year, then 50 mg QOD	Until progression	5-year PFS 57 (Thal) vs 44% (no treatment) *P*=0.0005	68 vs 65% *P*=0.04;
Morgan *et al.*^25^	820	Thal vs no Thal	Until progression	22 vs 15 months *P*<0.0001	60 months in both the groups *P*=0.70
Lokhorst *et al.*^26^	556	Thal 50 mg vs interferon 3 million IU TIW	Until progression	34 vs 25 months *P*<0.001 HR=0.67, 95% CI=0.55–0.82	73 vs 63 months *P*=0.77 HR=0.96, 95% CI 0.74–1.23
Stewart *et al.*^27^	332	Thal/Pred vs no treatment	4 years or until progression	4-year PFS 32 vs 14% HR=0.56 *P*<0.0001	4-year OS 68 vs 60% HR=0.77 *P*=0.18
Maiolino *et al.*^28^	108	Dex vs Thal+Dex	12 months or until progression	2-year PFS 30 (D) vs 64% (TD) *P*=0.002 Median PFS 19 (D) vs 36 months (TD)	2-year OS 70 (D) vs 85% (TD) *P*=0.27

Abbreviations: CI, confidence interval; Dex, dexamethasone; IU, international units; OS, overall survival; PFS, progression-free survival; Pred, prednisone; QOD, every other day; TIW, three times weekly; Thal, thalidomide.

**Table 2:** Major studies of lenalidomide maintenance

*Study*	N*, age*	*Regimens*	*PFS*	*OS*	*Side effects*
Attal *et al.*^23^	614,<65 years	Len 10 mg × 3 months; increase to 15 mg until relapse	41 vs 23 months HR, 0.50, *P*< 0.0001	4 years (70%) in both	MC=hematological SPM: 3.1 per 100 pt years vs 1.2 per 100 pt years (*P*<0.002) in the Len group
McCarthy *et al.*^35^	460,<71 years	Len 5–15 mg daily, 100 days after autograft	Median: 46 vs 27 months (*P*<0.001) 3-year PFS: 58 vs 37% HR, 0.48, 95% CI, 0.36–0.63)	88% (95% CI, 84–93) vs 80%, HR, 0.62, 95% CI, 0.40–0.95), *P*=0.03	SPM: Len: Hem: 3.5%, ST: 4.3% Placebo: Hem: 0.4% ST: 2.2%
Palumbo *et al.*^36^	273,<65 years	Len 10 mg days 1–21 out of 28 days	Median PFS: 41.1 vs 21 months, HR, 0.47, 95% CI, 0.33–0.65, *P*<0.001	3-year OS: 88.0 vs 79.2% HR, 0.64, 95% CI, 0.36–1.15, *P*=0.14	MC=Grades 3–4 neutropenia No difference in incidence of SPM
Gay *et al.*^38^	389,<65 years	Len 10 mg days 1–21 plus prednisone 50 mg (LP) vs Len (L) 10 mg alone	LP=37.5 months (95% CI, 27.8–not evaluable) L=28.5 months (95% CI 22.5–46.5) HR, 0.84, 95% CI, 0.59–1.20, *P*=0.34	—	No difference in adverse events

Abbreviations: CI, confidence interval; Hem, hematological malignancies; HR, hazard ratio; Len, lenalidomide; MC, most common; OS, overall survival; PFS, progression-free survival; pt, patient; SPM, secondary primary malignancies; ST, solid tumor.

**Table 3:** Major studies of bortezomib maintenance

*Study*	N*, age*	*Regimens*	*PFS*	*OS*	*Side effects*
Sonneveld *et al.*^41^	827,<65 years	BTZ 1.3 mg/m^2^/2 weeks vs Thal 50 mg/day for 2 years	35 vs 28 months HR, 0.75, 95% CI, 0.61–0.90, *P*=0.002	5 year 61 vs 55% HR, 0.81, 95% CI, 0.63–1.05, *P*=0.11	PN: 5 vs 8% SPM: 3.1 per early discontinuation for toxicity: 11 vs 30%
Rosinol *et al.*^43^	386,<65 years	IFN-α 3MU 3 × /week vs Thal 100 mg/day vs Thal 100 mg/day+BTZ 1.3 mg/m^2^ on days 1, 4, 8 and 11 every 3 months for 3 years	2-year PFS: 49 vs 63 vs 78%, months (*P*=0.01)	NS	

Abbreviations: 3MU, 3 million units; BTZ, bortezomib; CI, confidence interval; HR, hazard ratio; IFN, interferon; NS, not significant; OS, overall survival; PFS, progression-free survival; PN, peripheral neuropathy; SPM, secondary primary malignancies; Thal, thalidomide.

**Table 4:** Ongoing maintenance studies after ASCT with newer agents

*ClinicalTrial.gov identifier*	*Phase*	*Arms*	
*Ixazomid-based maintenance*			
NCT02504359	I	Ixazomib	2 years
NCT02253316	II	Lenalidomide vs ixazomib	3 years
NCT02619682	II	Alternating lenalidomide+ixazomib	2 years
NCT02168101^a^	II	Ixazomib	6 months
NCT01936532	II	Ixazomib	1 year
NCT02181413	III	Ixazomib vs placebo	2 years
NCT02406144	III	Lenalidomide vs lenalidomide+ixazomib	2 years in MRD– 5 years in MRD+
*Carfilzomid-based maintenance*			
NCT02315716	II	Carfilzomib	18 months
NCT01816971^a^	II	Lenalidomide+carfilzomib+dexamethasone	10 months
NCT02659293	III	Lenalidomide vs lenalidomide+carfilzomib+dexamethasone	NA
*Elotuzumab-based maintenance*			
NCT02655458	I	Lenalidomide+elotuzumab	1 year
NCT02843074	II	Elotuzumab	2 years
NCT02420860	II	Lenalidomide+elotuzumab	NA
NCT02375555	II	Lenalidomide+bortezomib+elotuzumab	NA
NCT02495922	III	Lenalidomide vs lenalidomide+elotuzumab	2 years
*Daratumumab-based maintenance*			
NCT02541383	III	Daratumumab vs observation	2 years
*Vorinostat-based maintenance*			
NCT00729118	I	Lenalidomide+vorinostat	Until progression
NCT00839956	II	Bortezomib+vorinostat	1 year
NCT01554852^b^	III	Observation vs lenalidomide vs lenalidomide+vorinostat	Until progression
*Panobinostat-based maintenance*			
NCT01440582	I	Lenalidomide+panobinostat	NA
NCT02145715	I–II	Panobinostat	1 year
NCT02722941	II	Panobinostat	NA
NCT02720510	II	Lenalidomide Lenalidomide+panobinostat	3 years
NCT02802163	II	Lenalidomide+panobinostat	Until progression 1 year

Abbreviations: ASCT, autologous stem cell transplant; MRD, minimal residual disease; NA, not available.

a Planned posttransplant intervention is <1 year; however, this intervention is referred as maintenance in the clinical trial.

b Maintenance will be evaluated after autologous hematopoietic stem cell transplantation and in non-transplanted patients.
